# P-1585. Safety and Efficacy of BCG Vaccination in COVID-19 Prevention: A Systematic Review and Meta-Analysis

**DOI:** 10.1093/ofid/ofaf695.1764

**Published:** 2026-01-11

**Authors:** Ahmed Hosney Nada, Ismail A Ibrahim, Asmaa Zakria Alnajjar, Talya Mansour, Tuğba Saka, Bareera Tanveer Malik

**Affiliations:** Faculty of Medicine, Benha University, Egypt, Quesna, Al Minufiyah, Egypt; Fenerbahce University, Faculty of Health Sciences, Istanbul, Istanbul, Turkey; Faculty of Medicine, Al-Azhar University, Gaza, Palestine, Gaza, Not Applicable, Palestinian Territories; Beirut Arab University, Beirut, Beyrouth, Lebanon; İstinye üniversitesi, Turkey, Istanbul, Turkey; Shaikh Khalifa Bin Zayed Al Nahyan Medical and Dental College, Sargodha, Punjab, Pakistan

## Abstract

**Background:**

Despite the availability of multiple COVID-19 vaccines, challenges remain in prevention and control. This systematic review and meta-analysis assess the safety and efficacy of BCG vaccination in COVID-19 prevention.

**Figure:**
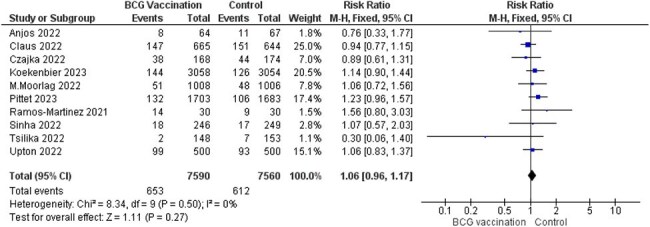

**Figure:**
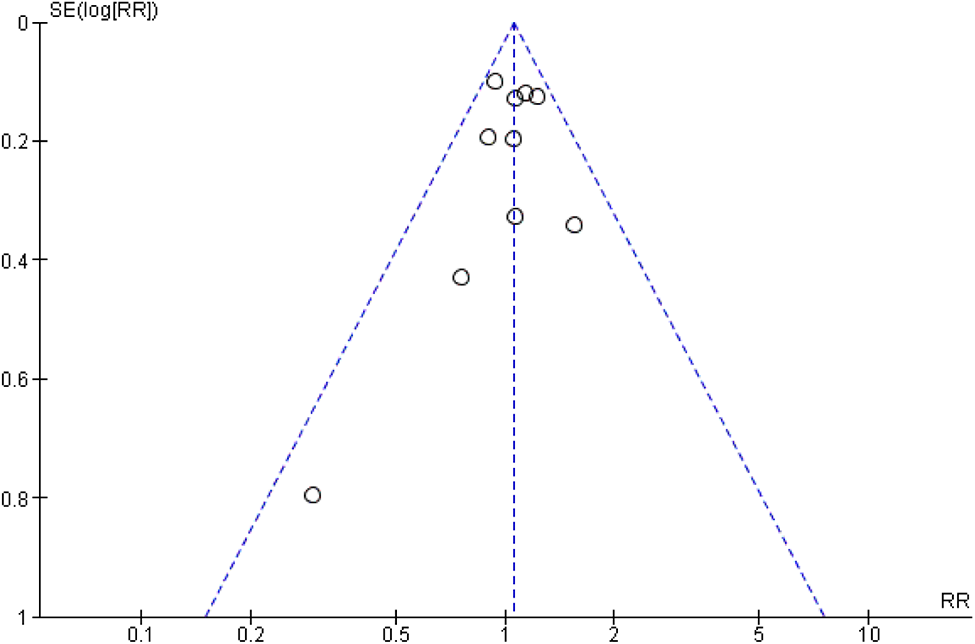

**Methods:**

A comprehensive search was conducted in PubMed, Cochrane, Scopus, Web of Science, and Embase until March 2024. Primary outcomes included COVID-19 infection rate, mortality, hospitalization rate, and ICU admission. Secondary outcomes focused on adverse events.

**Figure:**
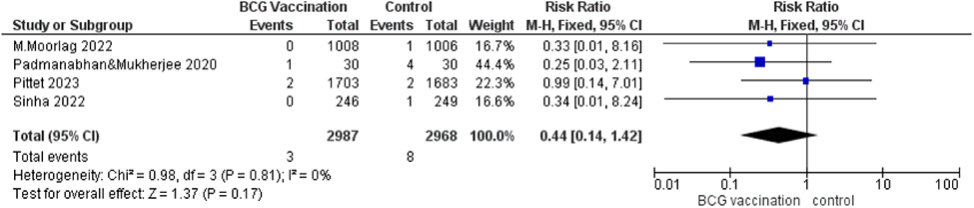

**Figure:**
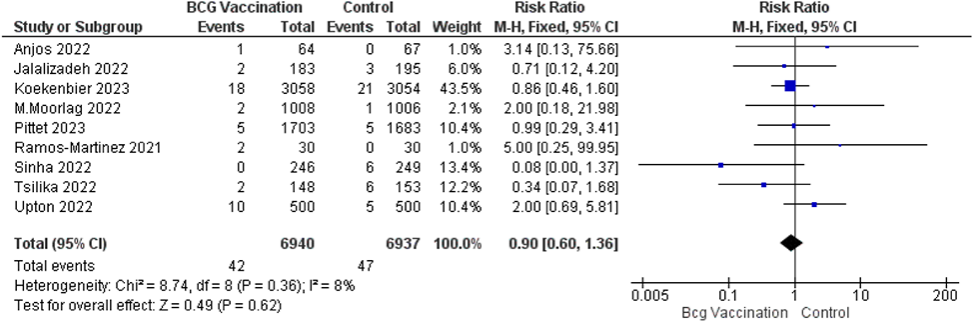

**Results:**

Fifteen RCTs with 16,347 participants were included. BCG vaccination did not significantly reduce COVID-19 infection (RR = 1.06, 95% CI [0.96–1.17], p = 0.27), ICU admission (RR = 0.44, 95% CI [0.14–1.42], p = 0.17), or hospitalization rate (RR = 0.90, 95% CI [0.60–1.36], p = 0.62). There were fewer deaths in the BCG group (RR = 0.52, 95% CI [0.25–1.08], p = 0.08). Regarding adverse events, BCG vaccination was strongly associated with erythema and may slightly increase cough risk, while other systemic symptoms (nausea, muscle/joint pain, sore throat) showed no significant association.

**Conclusion:**

BCG vaccination does not significantly reduce the risk of COVID-19 infection, hospitalization, or ICU admission. While there is a potential reduction in mortality, the result is not statistically significant. Adverse event analysis confirms a strong association with erythema and a slight increase in cough risk, but no consistent association with other systemic symptoms. These findings suggest that BCG vaccination does not provide substantial protection against COVID-19 but is generally well tolerated.

**Disclosures:**

All Authors: No reported disclosures

